# Molecular and Phylogenetic Characterisation of a Highly Divergent Novel Parvovirus (Psittaciform Chaphamaparvovirus 2) in Australian *Neophema* Parrots

**DOI:** 10.3390/pathogens10121559

**Published:** 2021-11-30

**Authors:** Subir Sarker

**Affiliations:** Department of Physiology, Anatomy and Microbiology, School of Life Sciences, La Trobe University, Bundoora, VIC 3086, Australia; s.sarker@latrobe.edu.au

**Keywords:** parvovirus, chaphamaparvovirus, phylogenetics, metagenomics, evolution

## Abstract

Parvoviruses under the genus *Chaphamaparvovirus* (subfamily *Hamaparvovirinae*) are highly divergent and have recently been identified in many animals. However, the detection and characterisation of parvoviruses in psittacine birds are limited. Therefore, this study reports a novel parvovirus, tentatively named psittaciform chaphamaparvovirus 2 (PsChPV-2) under the genus *Chaphamaparvovirus*, which was identified in Australian *Neophema* birds. The PsChPV-2 genome is 4371 bp in length and encompasses four predicted open-reading frames, including two major genes, a nonstructural replicase gene (NS1), and a structural capsid gene (VP1). The NS1 and VP1 genes showed the closest amino acid identities of 56.2% and 47.7%, respectively, with a recently sequenced psittaciform chaphamaparvovirus 1 from a rainbow lorikeet (*Trichoglossus moluccanus*). Subsequent phylogenetic analyses exhibited that the novel PsChPV-2 is most closely related to other *chaphamaparvoviruses* of avian origin and has the greatest sequence identity with PsChPV-1 (60.6%). Further systematic investigation is warranted to explore the diversity with many avian-associated parvoviruses likely to be discovered.

## 1. Introduction

Parvoviruses are small, nonenveloped, linear, single-stranded DNA (ssDNA) molecules of 4–6 kb in length. They encode two gene cassettes: a nonstructural replicase gene (NS1) that encodes the enzymes required for replication and a capsid (VP1) gene encoding structural proteins [1,2]. Viruses within the family *Parvoviridae* are currently grouped into three phylogenetically defined subfamilies: *Parvovirinae* (10 genera), which contains viruses that infect mammals, birds, and reptiles; *Densovirinae* (11 genera), which comprises viruses infecting invertebrate hosts, including insects, crustacea, and echinoderms; and *Hamaparvovirinae*, a recently established taxon that contains viruses identified in vertebrate (two genera) and invertebrate (three genera) hosts [1,3,4]. Among the 10 genera within the subfamily *Parvovirinae*, the genus *Chaphamaparvovirus* has been detected in the faecal materials of chickens, turkeys, rats, pigs, and *Eidolon helvums*, and various tissue samples including serums, rectal swabs, nasal swabs, and lung lavages sourced from pigs [5,6,7,8]. Additionally, a novel avian chaphamaparvovirus was recently detected in brain, liver, and heart tissues collected from rainbow lorikeet (*Trichoglossus moluccanus*) [9]. However, it is currently unknown whether these viruses are associated with known diseases.

Very little is known about the parvoviruses that may harbour in psittacine birds, and almost nothing is known about parvoviruses in psittacine birds under the genus *Neophema*, an Australian genus with six parrot species, including one of the most critically endangered: the orange-bellied parrot (*Neophema chrysogaster*). Therefore, this study aims to characterise a novel parvovirus identified in the faecal materials collected from elegant parrots (*Neophema elegans*) and scarlet-chested parrots (*Neophema splendida*).

## 2. Results and Discussion

### 2.1. Genome of PsChPV-2

**Figure 1:**
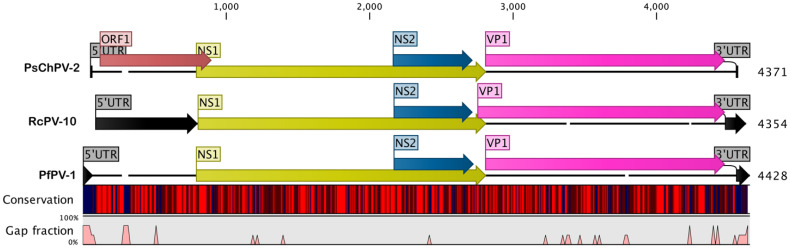
A schematic illustration of the selected avian chaphamaparvoviruses. A schematic map of the psittaciform chaphamaparvovirus 2 (PsChPV-2, GenBank accession No. MZ364297) compared to the parvoviridae sp. isolate 10 (RcPV-10, GenBank accession No. KY312549) and the peafowl parvovirus 1 (PfPV-1, GenBank accession No. MK988619), using the CLC Genomic Workbench (version 9.5.4, CLC bio, a QIAGEN Company, Hilden, Germany). The arrows symbolise chaphamaparvovirus genes and open-reading frames (ORFs) predicted to code for proteins, indicating their transcription direction. Each gene or ORF is colour-coded, as indicated by the different colours. The middle graph represents the sequence conservation between the aligned PsChPV-2, RcPV-10, and PfPV-1 sequences at a given coordinate at each position in the alignment. The colour gradient reflects the conservation of that position in the alignment. Red presents 100% conservation across all three viruses, black is 50% conserved regions, and blue is less than 50% conserved regions. The bottom graph represents the gap fraction between the aligned sequences.

The fully assembled psittaciform chaphamaparvovirus 2 (PsChPV-2) genome is a linear, single-stranded DNA (ssDNA) molecule 4371 nucleotides (nt) in length and has a basic organisation, similar to previously described members of the *Parvoviridae* family (Figure 1). Like most parvoviruses, the PsChPV-2 genome is flanked on the 5′ and 3′ ends by 17 nt inverted repeat sequences, with the coordinates of 1–17 sense orientation and 4355–4371 antisense orientation. The PsChPV-2 genome contains 34.3% A, 19.4% G, 24.6% T, and 22.1% C, with an A + T content of 58.9% and a C + G content of 41.6%. The overall genomic organisation of PsChPV-2 is similar to other parvoviruses, with two major predicted open-reading frames (ORFs) that typically contain a replication initiator protein, called NS1, and a viral capsid (VP1; Figure 1).

### 2.2. Comparative Analyses of PsChPV-2

A comparative analysis of the protein sequences encoded by the predicted ORFs, using BLASTX and BLASTP, recognised a significant protein sequence similarity (E value  ≤  10^−5^) for all four ORFs (Figure 1) according to the BLAST database. The 5′ ORF1 is 687 nt long, and a BLAST search using the putative amino acid sequence revealed 59.4% protein, similar to the hypothetical MAG protein of the Phoenicopteridae parvo-like hybrid virus (GenBank accession No. QTE03742.1). PsChPV-2 nonstructural (NS) protein one (NS1) ORF is 2016 nt long and shows a relatively low sequence similarity compared to other parvoviruses’ isolates (Table 1 and Supplementary Figure S1). At the amino acid level, PsChPV-2 NS1 protein exhibited 37.1% to 56.2% amino acid identity compared to the other *Parvoviridae* NS1, with the greatest similarity to the recently identified psittaciform chaphamaparvovirus 1 (protein similarity 56.2.%, GenBank accession No. MT457858). Similar to other parvoviruses, the complete NS1 gene of PsChPV-2 is 671 amino acids in length and encodes helicase, including the conserved ATP- or GTP-binding Walker A loop (GPxNTGKT/S; _317_**GP**S**NTGKS**_324_), Walker B (xxxWEE; _355_IGV**WEE**_360_), Walker B’ (KQxxEGxxxxxPxK; _372_**KQ**VL**EG**MQTSI**P**I**K**_385_), and Walker C (PxxxTxN; _396_**P**III**T**S**N**_402_) aa motifs. In addition, the NS1 protein contains two conserved replication initiator (endonuclease) motifs, xxHuHxxxx (IF_108_**H**V**H**_110_AMLQ) and YxxK (_166_**Y**LM**K**_169_) (conserved amino acids are indicated in bold letters, and u indicates a hydrophobic residue) (Supplementary Figure S1) [10,11].

The major 3ʹ ORF is 1626 nt long and encodes proteins such as the *Parvoviridae* capsid protein VP1. At the amino acid level, PsChPV-2 VP1 protein exhibits 35.2% to 47.7% amino acid identities similar to the other *Parvoviridae* VP1, with the greatest similarity to psittaciform chaphamaparvovirus 1 (protein similarity 47.7%, GenBank accession No. MT457858; Table 1 and Supplementary Figure S2). The PsChPV-2 genome also contains a 555 nt long ORF, which was shown to be homologous to the NS2 protein of the peafowl parvovirus 2 (protein similarity 51.4%, query coverage 98% and E-value: 2.0 × 10^−55^, GenBank accession No. QGJ83205.1 [12]).

According to the complete genome sequence analysis, the novel PsChPV-2 genome is most closely related to psittaciform chaphamaparvovirus 1 (PsChPV-1; nucleotide similarity 60.6%), followed by Avian parvoviridae sp. isolate 9 (RcPV-9; nucleotide similarity 55.6%), peafowl parvovirus 1 (PfPV-1; 53.8%), parvoviridae sp. isolate 10 (RcPV-10; nucleotide similarity 53.5%), galliform chaphamaparvovirus 1 (GaChV-1; 52.7%), and chicken chapparvovirus 1 (CchV-1; 50.0%; Table 1).

### 2.3. Evolutionary Relationships of PsChPV-2

**Figure 2:**
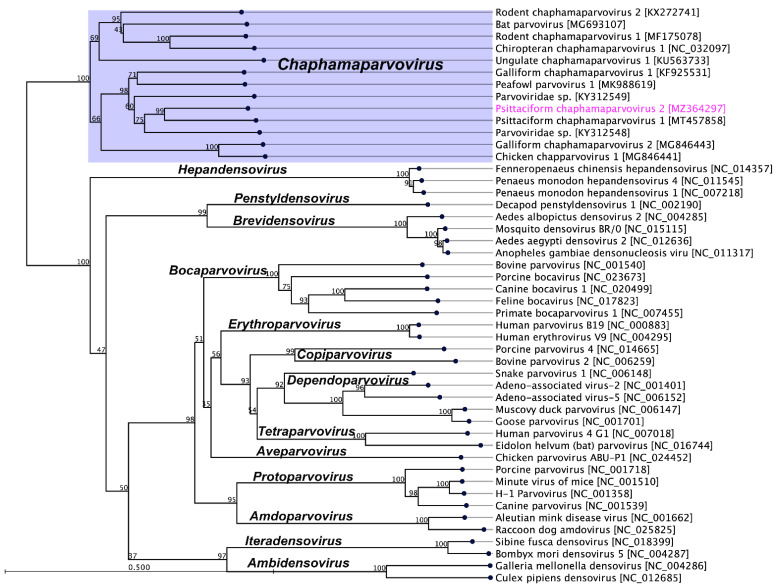
The phylogenetic tree shows the possible evolutionary relationship of psittaciform chaphamaparvovirus 2 with other selected parvoviruses. The maximum likelihood (ML) tree was constructed using the amino acid sequences of complete nonstructural protein 1 (NS1). The numbers on the left show bootstrap values as percentages and the labels at the branch tips refer to the original parvoviruses’ species names, followed by the GenBank accession numbers in parentheses. The clade correspondence to the genus *Chaphamaparvovirus* has a purple background, and the psittaciform chaphamaparvovirus 2 sequenced in this study is shown in pink.

**Figure 3:**
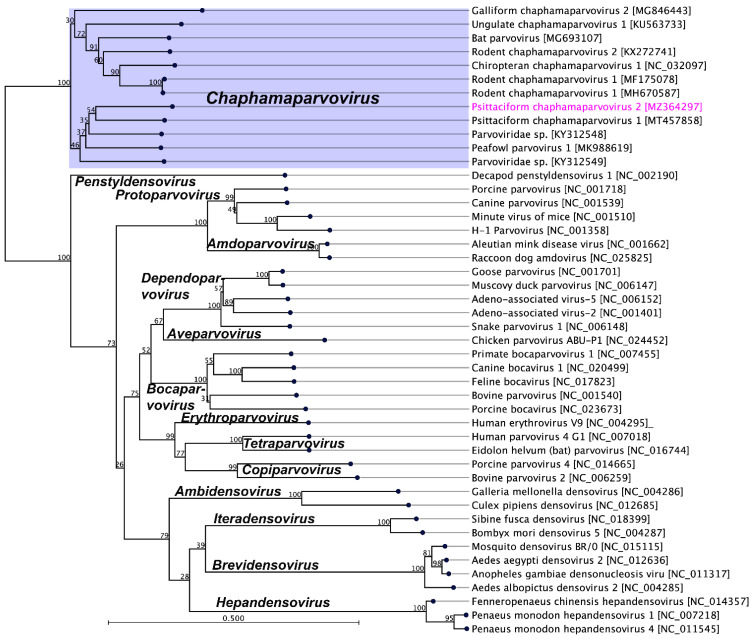
The phylogenetic tree shows the possible evolutionary relationship of psittaciform chaphamaparvovirus 2 with other selected parvoviruses. The maximum likelihood (ML) tree was constructed by using the amino acid sequences of the complete capsid protein (VP1). The numbers on the left show bootstrap values as percentages and the labels at branch tips refer to the original parvoviruses’ species names, followed by the GenBank accession numbers in parentheses. The clade correspondence to the genus *Chaphamaparvovirus* has a purple background, and the Psittaciform chaphamaparvovirus 2 sequenced in this study is shown in pink.

Members of the parvovirus subfamilies are distinguished mainly by their vertebrate or invertebrate host range, but this organisation is also strongly supported by a phylogenetic analysis based on the amino acid sequence of the viral replication initiator protein [4]. Accordingly, a phylogenetic analysis based on parvoviral replication initiator protein (NS1) sequences clearly support the addition of the newly sequenced PsChPV-2 to the genus *Chaphamaparvovirus*. In the resulting maximum likelihood (ML) tree, the sequenced PsChPV-2 clustered in a distinct subclade with other parvoviruses such as psittaciform chaphamaparvovirus 1, galliform chaphamaparvovirus 1 and 2, peafowl parvovirus, and two others avian parvoviridae sp. (Figure 2). Using the same set of NS1 protein sequences, we found that the maximum interlineage sequence identity values between the novel PsPV1 and other parvoviruses are 56.2% (PsChPV-2 vs. PsChPV-1), 49.7% (PsChPV-2 vs. RcPV-9), 49.2% (PsChPV-2 vs. PfPV-1), and 43.9% (PsChPV-2 vs. GaChV-1), which mirror the phylogenetic position of this novel PsChPV-2. Furthermore, the ML tree based on the protein sequences of the VP1 gene demonstrated similar tree topologies for the representative parvovirus species, where PsChPV-2 is positioned in a distinct subclade with other chaphamaparvoviruses of avian origin (Figure 3).

## 3. Materials and Methods

### 3.1. Sampling and Ethical Approval

In 2020, four fresh faecal samples were collected from two different species, the elegant parrot (*Neophema elegans*) and the scarlet-chested parrot (*Neophema splendida*), housed in the La Trobe Animal Research and Teaching Facility for the Parrot Genome Sequencing Project. Samples were stored at −80 °C within one hour of their collection and kept at that temperature until further processing. Bird sampling was obtained following approved guidelines set by the Australian Code of Practice for the Care and Use of Animals for Scientific Purposes and approved by the La Trobe University Animal Ethics Committee (research permit number AEC19035) and the Department of Environment, Land, Water and Planning (permit number 10009300).

### 3.2. Virus Enrichment and Virus Nucleic Acid Extraction

Eliminating likely impurities, such as host cells, bacteria, food particles, and free nucleic acids, from the faecal samples, followed by virus particle enrichment, was performed under the stated methods [16,17], with minor variations. Briefly, the faecal materials were aseptically resuspended and vigorously homogenised in sterile phosphate-buffered saline (PBS; 1:10) and centrifuged at 2500× *g* for 90 min at 4 °C. The supernatant was filtered using a 0.80 μm syringe filter, and the filtrate was processed downstream. The samples were then ultracentrifuged at 178,000× *g* and 30 psi for one hour at 4 °C using a Hitachi Ultracentrifuge CP100NX (Hitachi Koki Co., Ltd., Tokyo, Japan). The supernatant was discarded and the pellet was suspended in 130 µL of sterile PBS. The filtrates were then nuclease-treated using 2 µL of benzonase nuclease (25–29 U/µL, purity  >  90%, Millipore) (Merck KGaA, Darmstadt, Germany) and 1 µL of micrococcal nuclease (2,000,000 gel units/mL; New England Biolabs, Ipswich, MA, USA) and incubated at 37 °C for two hours. The nuclease reaction was stopped by adding 3 µL of 500 mM ethylenediaminetetraacetic acid (EDTA). The viral nucleic acids were extracted using a QIAamp Viral RNA Mini Kit (Qiagen, Valencia, CA, USA) without carrier RNA, which allowed the simultaneous extraction of viral DNA and RNA. The quantity and quality of the isolated nucleic acids were determined using a Nanodrop and an Agilent Tape Station (Agilent Technologies, Mulgrave, VIC, Australia) and the Genomic Platform, La Trobe University.

### 3.3. Next-Generation Sequencing

Before library construction, the quantity and quality of the extracted nucleic acids were checked using a Qubit dsDNA high-sensitivity assay kit with a Qubit Fluorometer v3.0 (Thermo Fisher Scientific, Waltham, MA, USA). The library construction was performed as a pool that contained four samples using the Illumina DNA Prep (Illumina, San Diego, CA, USA) as per kit instructions, starting with 250 ng of DNA as measured by a Qubit Fluorometer v3.0 (Thermo Fisher Scientific, Waltham, MA, USA). The quality and quantity of the prepared library were assessed by the Australian Genome Research Facility (AGRF), Melbourne, Australia. The prepared library was normalised and pooled in equimolar quantities. The quality and quantity of the final pooled library were further assessed as described above before sequencing by the facility. According to the manufacturer’s instructions, cluster generation and sequencing of the pooled library were performed with read lengths of 150 bp paired-end on the Illumina^®^ NovaSeq chemistry.

### 3.4. Bioinformatic Analyses

The raw sequence reads (52.1 million) were used to assemble the complete genome of PsChPV-2 as previously stated [18,19,20,21,22,23,24] using CLC Genomics Workbench (version 9.5.4). An initial quality assessment for all raw reads was produced, which were preprocessed to eliminate ambiguous base calls, and poor-quality reads and then trimmed to remove the Illumina adapter sequences. The clean sequence reads were mapped against the chicken genome (*Gallus*, GenBank accession No. NC_006088) to filter out likely host-DNA contamination. In addition, reads were further mapped to the *Escherichia coli* bacterial genomic sequence (GenBank accession No. U00096) to eliminate potential bacterial contamination. Unmapped reads were used as input data for de novo assembly using SPAdes assembler (version 3.10.1; [25] under the ‘careful’ parameter at La Trobe Institute for Molecular Science–High-Performance Computer (LIMS-HPC) system. This resulted in a single contig found to be a parvovirus based on the BLAST search [26]. Clean raw reads (44.3 million) were mapped back to the novel PsChPV-2 genome, which resulted in an average coverage of 692.64×, where a total of 21,488 reads were mapped to PsChPV-2. The detected complete genome of the parvovirus was annotated using Geneious software (version 10.2.2, Biomatters, New Zealand), where parvoviridae sp. isolate 10 (RcPV-10, GenBank accession No. KY312549) and peafowl parvovirus 1 (PfPV-1, GenBank accession No. MK988619) were used as reference guidelines. Similarly, BLAST searches were performed on the predicted ORFs and annotated as potential genes if the predicted ORFs showed significant sequence identities to known viral or cellular genes (E-value threshold of 1 × 10^−5^) [26].

### 3.5. Comparative Genomics and Phylogenetic Analyses

The genomic features of the novel psittaciform chaphamaparvovirus 2 (PsChPV-2) genome were visualised using Geneious (version 10.2.2). Sequence similarity percentages between representative viruses were determined using tools available in Geneious (version 10.2.2). For phylogenetic analyses, demonstrative parvoviral gene sequences were downloaded from GenBank and trees were constructed using CLC Genomics Workbench (version 9.5.4). The amino acid sequences of protein-coding genes of the selected genes were aligned using the MAFTT L-INS-I algorithm implemented in Geneious (version 7.388) [27]. Phylogenetic analyses for protein sequences were performed using the WAG substitution model, with 1000 bootstrap replicates in CLC Genomics Workbench (version 9.5.4).

## 4. Conclusions

This study reports the genomic characterisation of PsChPV-2, which was sequenced from the faecal materials of Australian *Neophema* birds. The novel PsChPV-2 genome recovered in this study is significantly divergent compared to other sequenced parvoviruses and only shows a 60.6% nucleotide identity with a recently sequenced avian chaphamaparvovirus. Considering the overall genome architecture and nucleotide identity, PsChPV-2 appears to represent a novel species, tentatively designated psittaciform chaphamaparvovirus 2 within the genus *Chaphamaparvovirus* and family *Parvoviridae*. Additional studies screening for parvoviruses in wild and captive Australian parrots and generating the whole parvovirus genome sequence of native Australian psittacine species will be required to better understand the parvovirus’s diversity and evolution.

## Figures and Tables

**Table 1:** A comparative analysis of representative chaphamaparvovirus species against psittaciform chaphamaparvovirus 2 (PsChPV-2) based on complete genome and conserved gene sequences.

Chapparvoviruses	Host	GenBank Accession Numbers	Abbreviation	Genome Identity (%)	NS1			VP1	References
					nt	aa	nt	aa	
Psittaciform chaphamaparvovirus 2	*Neophema elegans/ N. splendida*	MZ364297	PsChPV-2						This study
Psittaciform chaphamaparvovirus 1	*Trichoglossus moluccanus*	MT457858	PsChPV-1	60.6	65.3	56.2	54.6	47.7	[9]
Peafowl parvovirus 1	*Pavo cristatus*	MK988619	PfPV-1	53.8	59.9	49.2	49.8	43.4	[12]
Galliform chaphamaparvovirus 1	*Meleagris gallopavo*	KF925531	GaChV-1	52.7	57.9	43.9			[5]
Chicken chapparvovirus 1	*Gallus gallus*	MG846441	CChV-1	50.0	50.2	36.4			unpublished *
Galliform chaphamaparvovirus 2	*Gallus gallus*	MG846443	GaChV-2	45.8	48.8	34.5	45.6	43.7	unpublished *
Parvoviridae sp.	*Grus japonensis*	KY312548	RcPV-9	55.6	59.4	49.7	53.1	40.5	unpublished *
Parvoviridae sp.	*Grus japonensis*	KY312549	RcPV-10	53.5	59.5	46.8	52.8	39.1	unpublished *
Rodent chaphamaparvovirus 1	*Mus musculus*	MF175078	RoChV-1	45.4	47.6	36.7	47.0	39.7	[13]
Chiropteran chaphamaparvovirus 1	*Desmodus rotundus*	NC_032097	ChiCPV-1	46.0	49.2	34.0	47.0	39.6	[10]
Bat parvovirus	*Eidolon helvum*	MG693107	BtPV	46.4	48.5	33.4	46.0	39.7	[14]
Rodent chaphamaparvovirus 2	wild rat	KX272741	RoChV-2	44.2	47.8	35.8	43.5	37.7	[15]
Ungulate chaphamaparvovirus 1	swine	KU563733	UChPPV-1	41.1	43.4	31.7	42.0	35.2	[7]

* = publicly available in GenBank; aa, amino acid; nt, nucleotides.

## Data Availability

The complete genome sequence of novel PsChPV-2 was deposited in the NCBI GenBank under accession number MZ364297. Raw sequencing data from this study were deposited in the NCBI Sequence Read Achieve (SRA) under accession number SRR15309349 (BioProject ID: PRJNA750905; BioSample accession: SAMN20500864) (http://www.ncbi.nlm.nih.gov/sra/, accessed on 25 August 2021).

## Supplementary Materials

The following are available online at https://www.mdpi.com/article/10.3390/pathogens10121559/s1, Figure S1: Alignment of the amino acid sequences of the complete *NS1* gene under the genus *Chaphamaparvovirus* using MAFFT in Geneious (version 10.2.2) to show the genetic variation between psittaciform chaphamaparvovirus 2 (PsChPV-2) and other selected parvoviruses. PsChPV-2 is highlighted with a yellow background, Figure S2: Alignment of the amino acid sequences of the complete *VP1* gene under the genus *Chaphamaparvovirus* using MAFFT in Geneious (version 10.2.2) to show the genetic variation between psittaciform chaphamaparvovirus 2 (PsChPV-2) and other selected parvoviruses. PsChPV-2 is highlighted with a yellow background.
