# Supplementary material for: Molecular and Phylogenetic Characterisation of a Highly Divergent Novel Parvovirus (Psittaciform Chaphamaparvovirus 2) in Australian Neophema Parrots
Source: Pathogens. 2021 Nov 30;10(12):1559. doi: 10.3390/pathogens10121559 (PMC8706300; doi:10.3390/pathogens10121559)
Supplement: Supplementary file 1 [file pathogens-10-01559-s001.zip › pathogens-1478062-supplementary.pdf]

pathogens MDPI

Supplementary File

Molecular and phylogenetic characterisation of a highly divergent novel parvovirus (Psittaciform chaphamaparvovirus 2) in Australian *Neophema* parrots

Subir Sarker[a*]

[a] Department of Physiology, Anatomy and Microbiology, School of Life Sciences, La Trobe University, Bundoora, VIC 3086, Australia

[*]**Author for correspondence:** Dr Subir Sarker, Department of Physiology, Anatomy and Microbiology, School of Life Sciences, La Trobe University, Bundoora, VIC 3086, Australia, email: s.sarker@latrobe.edu.au; phone: +61 3 9479 2317; fax: +61 3 9479 1222.

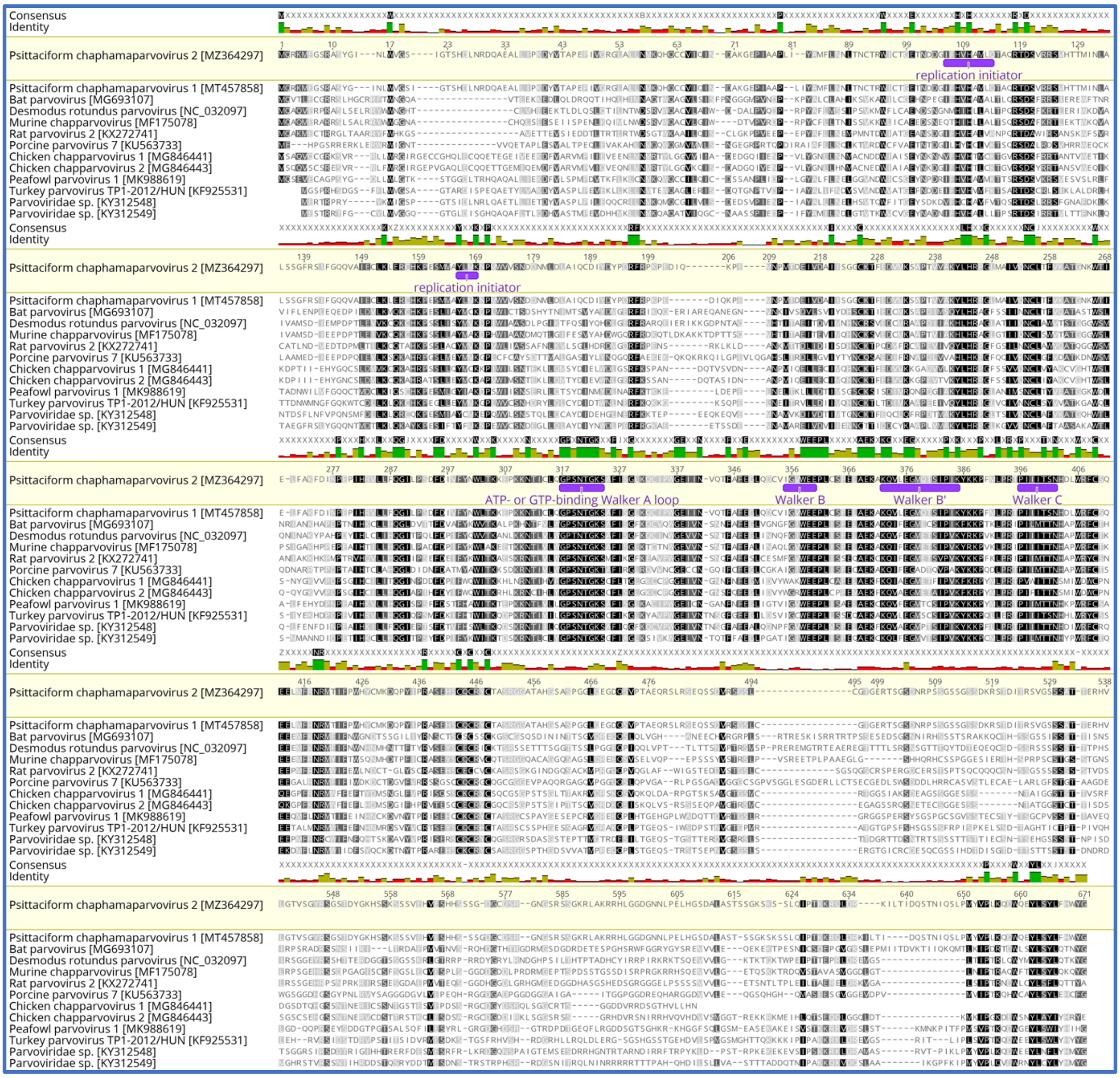

Figure S1. Alignment of the amino acid sequences of the complete NS1 gene under the genus *Chaphamaparvovirus* using MAFFT in Geneious (version 10.2.2) to show the genetic variation between Psittaciform chaphamaparvovirus 2 (PsChPV-2) and other selected parvoviruses. PsChPV-2 was highlighted with yellow background. The top graph represents the mean pairwise sequence identity over all pairs in the column (green: 100% identity; mustard: ≥30% and <100% identity; red: <30% identity).

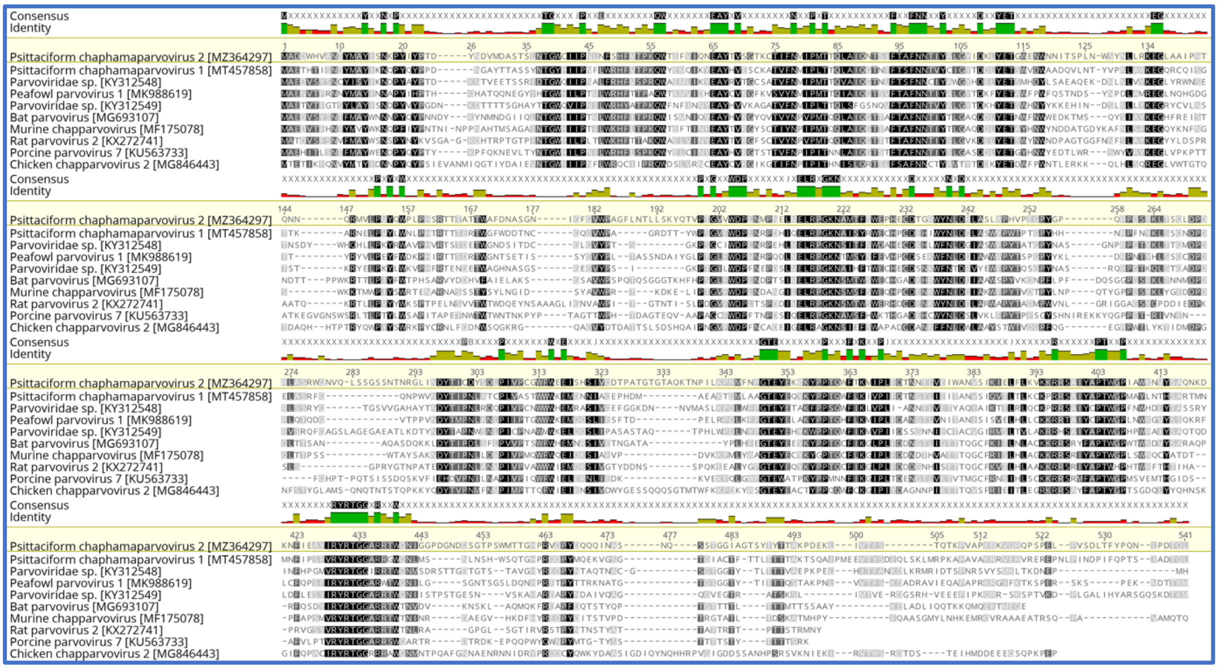

Figure S2. Alignment of the amino acid sequences of the complete VP1 gene under the genus *Chaphamaparvovirus* using MAFFT in Geneious (version 10.2.2) to show the genetic variation between Psittaciform chaphamaparvovirus 2 (PsChPV-2) and other selected parvoviruses. PsChPV-2 was highlighted with yellow background. The top graph represents the mean pairwise sequence identity over all pairs in the column (green: 100% identity; mustard: ≥30% and <100% identity; red: <30% identity).
